# Cancer Patients during COVID-19 Pandemic: A Mini-Review

**DOI:** 10.3390/healthcare11020248

**Published:** 2023-01-13

**Authors:** Maryam Linjawi, Hira Shakoor, Serene Hilary, Habiba I. Ali, Ayesha S. Al-Dhaheri, Leila Cheikh Ismail, Vasso Apostolopoulos, Lily Stojanovska

**Affiliations:** 1 Department of Nutrition and Health, College of Medicine and Health Sciences, United Arab Emirates University, Al Ain P.O. Box 15551, United Arab Emirates; 2 Department of Clinical Nutrition and Dietetics, College of Health Sciences, Research Institute of Medical and Health Sciences (RIMHS), University of Sharjah, Sharjah P.O. Box 27272, United Arab Emirates; 3 Nuffield Department of Women’s and Reproductive Health, University of Oxford, Oxford, OX3 9DU, UK; 4 Institute for Health and Sport, Victoria University, Melbourne, VIC 3030, Australia

**Keywords:** cancer, COVID-19, immunity, vaccine, patient care

## Abstract

Since its emergence, coronavirus disease 2019 (COVID-19) has affected the entire world and all commerce and industries, including healthcare systems. COVID-19 adversely affects cancer patients because they are immunocompromised. Increased COVID-19 infection and shortage of medical supplies, beds and healthcare workers in hospitals affect cancer care. This paper includes a description of the existing research that shows the impact of COVID-19 on the management of cancer patients. Aged people with various chronic conditions such as cancer and comorbidities face more challenges as they have a greater risk of disease severity. COVID-19 has affected care delivery, including patient management, and has been responsible for increased mortality among cancer patients. Cancer patients with severe symptoms require regular therapies and treatment; therefore, they have a higher risk of exposure. Due to the risk of transmission, various steps were taken to combat this disease; however, they have affected the existing operational efficiency. Herein, we present the changing priorities during COVID-19, which also affected cancer care, including delayed diagnosis, treatment, and surgeries.

## 1. Introduction

COVID-19 infection caused by severe acute respiratory syndrome coronavirus 2 (SARS-CoV-2) is phenotypically diverse and impacts patients depending on their immunity. COVID-19 infections have affected individuals’ health status, increased the healthcare system’s burden, and affected the delivery of healthcare services. According to the World Health Organization, over 589 million people to date have contracted SARS-CoV-2, leading to over 6.4 million deaths [1]. The link between communicable diseases and cancer is well known; however, it is crucial to understand how COVID-19 affects cancer management. Cancer patients are at greater risk of COVID-19 complications and increased mortality [2].

According to the World Health Organization, cancer is one of the leading causes of death worldwide, with an estimated 10 million deaths in 2020, accounting for one in six deaths [1]. Cancer patients are more vulnerable to infection compared to the general population. One reason is that cancer patients are immunosuppressed through tumor malignancy and anti-tumor treatments. Moreover, these individuals also present poor health status and often have other coexisting medical conditions. Hence, a cancer patient who suffers from COVID-19 may lead to a poor prognosis and increased mortality risk [3,4]. In addition, COVID-19 has affected all aspects of cancer care, such as diagnosis, treatment, and surgeries. 

COVID-19 can be an asymptomatic, mild, and sometimes severe disease [5]. Those with severe symptoms are often individuals with underlying pre-existing diseases, the elderly and those with low immune system status, such as cancer patients. These individuals can also develop severe health conditions such as respiratory failure, organ failure and other problems that cause mortality [6]. It has been reported that patients with cancer who have symptoms of COVID-19 require a greater level of intensive care; however, cancer has different subtypes that define the vulnerability [7]. Therefore, the impact of COVID-19 on the management of cancer patients could differ depending on the severity of the illness, stage and type of cancer. In addition, demographics and socioeconomic factors could affect an individual’s health differently. Considering the risk of COVID-19 to cancer patients, various changes have been introduced in cancer care management, including a multidisciplinary approach and digital or telehealth [8]. The objective of this mini-review was to present existing research on how COVID-19 adversely affected cancer patient care and what steps have been taken to modify the delivery of patient care during the pandemic. This narrative mini-review presents the relationship between cancer patients and the risk of COVID-19, mortality risk, and how it affects cancer care and management.

## 2. Methodology

Electronic databases such as PubMed, CINHAL and Google Scholar were searched with search terms: COVID-19, corona, SARS-CoV-2, coronaviruses, and cancer, malignancy, chemotherapy, cancer care, cancer management, immune, cytokines with filters identifying only studies published between April 2020–August 2022. Only articles published in English were included. The titles and abstracts were initially scanned to exclude any irrelevant studies. Exclusion criteria were defined as studies with no primary focus on cancer care management, if the full text was unavailable or if the identified search results were editorials, commentaries and book chapters. A total of 50 articles were finalized based on the abstracts and outcomes.

## 3. Inflammatory Biomarkers and COVID-19 Infection in Cancer Patients

**Figure 1:**
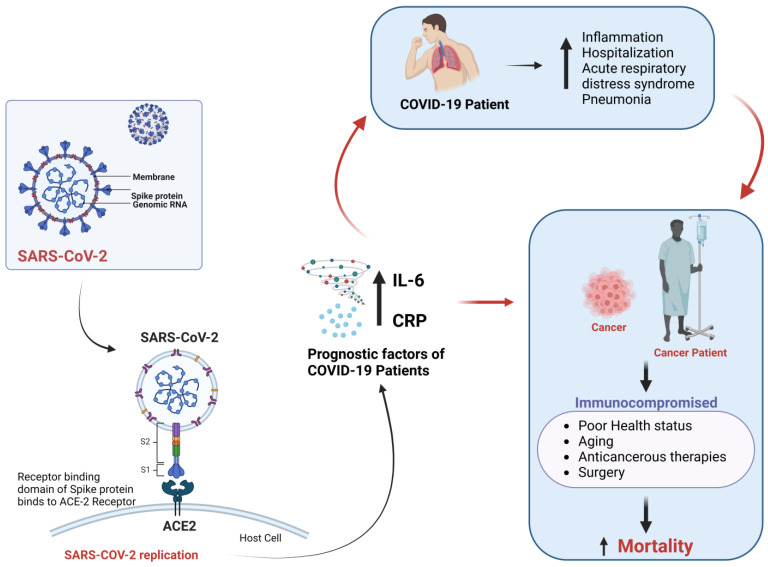
Effect of cancer and COVID-19 on immunity.

Cancer patients are generally immunocompromised, and COVID-19 infection may have worse outcomes than non-immunocompromised individuals. Specific biomarkers associated with severe COVID-19 illness in cancer patients include C-reactive protein (CRP) and interleukin (IL)-6. CRP and IL-6 levels may be helpful in the prognosis of COVID-19 infection (Figure 1). In this regard, CRP levels greater than 10 mg/dL were reported to be significantly associated with higher odds of COVID-19-related mortality (13.56, *p* = 0.03) in patients with leukemia, myeloma and lymphoma [9]. Similarly, IL-6 levels were also increased in severe COVID-19. Blocking IL-6 in cancer patients was previously reported to be beneficial when combined with other conventional therapies [10]. Tocilizumab, an anti-IL-6 antibody [11], can help decrease inflammation and mortality in COVID-19 subjects [12]. However, blocking this pathway could also increase the immunocompromised state and secondary infection [13].

In addition, the SARS-CoV-2 virus utilizes the angiotensin-converting enzyme 2 (ACE-2), a receptor on the surface of many cell types that provides an entry point for the virus [14], although other entry points have also been proposed. It was reported that the Toll-like receptor TLR4 significantly contributes to SARS-CoV-2 infectivity and pathogenesis. The SARS-CoV-2 S protein binds directly to the extracellular domain of TLR4, resulting in an aggressive inflammatory response in COVID-19 patients [15]. Further, SARS-CoV-2 S protein may utilize the immune receptors, including C-lectin type receptors (CLRs), neuropilin-1(NRP1) and non-immune receptor-like glucose-regulated protein 78 (GRP78) to enter the cell [16].

In addition, upregulation of ACE-2 expression on various cancer tissues may allow SARS-CoV-2 to bind with the receptor binding domain and enter host cells, eventually leading to increased mortality risk [17,18]. ACE-2 and receptor binding domain interaction could be a suitable target for antiviral drugs to treat COVID-19. Therefore, the allosteric modulation of ACE-2 might be a promising approach by inhibiting the interaction between ACE-2 and SARS-CoV-2 [19].

## 4. Cancer Types and COVID-19 Infection Severity

The progression and severity of COVID-19 are more rapid among cancer patients than non-cancer patients. This susceptibility is associated with the patient’s immunosuppressive state. Studies have highlighted that COVID-19 severity and progression mainly depend on the type of cancer. For instance, hematologic and lung cancer patients show the highest severity and death rates following COVID-19. In general, hematologic cancers lead to severe immunosuppression and, combined with COVID-19, exponentially increase patient mortality risk [20]. In a retrospective study, it was reported that pediatric cancer patients admitted to hospital for treatment had a higher risk of exposure to infection, and those with COVID-19 had greater mortality rates, particularly patients with hematologic malignancies [21].

Some solid tumors, such as lung and pancreatic cancer, require prompt diagnosis and treatment [22]. Lung cancer is associated with severe hypoxia, a notable feature of the tumor microenvironment in solid tumors. Cancer cells require more oxygen due to their rapid growth; thus, decreasing oxygen levels in regions of solid tumors results in necrosis and progression of the tumor. Hence, tumor cells become resistant to radiotherapy and chemotherapy [23,24]. Hypoxia is not the only condition of lung cancer that occurs during COVID-19. Studies have shown that several COVID-19-positive cancer patients require intensive-care, ventilator support, and oxygen therapy [25]. COVID-19 is similar to pneumonia and results in more pronounced respiratory failures [26]. Therefore, patients with lung cancer may be worse off due to pre-existing lung dysfunction [20]. Moreover, a study demonstrated that severe lymphopenia and high CRP levels are associated with the severity of hypoxemia and increased mortality rate in these patients [27].

## 5. Why Are Cancer Patients More Susceptible to COVID-19 Infection?

Cancer patients were the most susceptible individuals during the COVID-19 pandemic who had a higher risk of infection due to low immunity and associated therapeutic procedures such as chemotherapy, radiation, steroids and surgery. They rely heavily on medical resources and have higher needs for healthcare services, which makes them vulnerable to the pandemic [28]. Furthermore, cancer patients have an increased risk of COVID-19 due to their need to have regularly scheduled visits to the hospital, which further increases their risk of contact with other COVID-19 individuals. Similarly, cancer survivors and patients undergoing treatments have a higher risk of admission to the intensive care unit and an increased risk of mortality [29]. Therefore, cancer patients not only deal with disease and the risk of COVID-19 they also have increased anxiety and psychological outcomes.

Recent reports have identified that two groups have a higher risk of infection; individuals who completed cancer treatment and those currently undergoing treatment. Both groups of patients must receive health education to improve their safety [30,31]. A cancer patient who develops an infection such as COVID-19 and any other similar virus has symptoms similar to the general population; however, they tend to have haematological abnormalities such as anaemia, neutropenia or leukopenia. A range of clinical studies have focused on the relationship between cancer and COVID-19, and most of the studies concluded that patients with cancer had a poor prognosis during the pandemic, including increased hospitalization, bacterial infection and high mortality rates [31]. According to Sinha et al., cancer patients who received oncologic therapies such as any targeted or chemotherapy developed severe symptoms, and the mortality rate increased. Therefore, vaccination has been adopted as a strategic approach to reducing the risk for patients with cancer [32].

## 6. Increased Risk of Other Comorbidities Associated Cancer in COVID-19 Patients

Patients infected with the SARS-CoV-2 virus have elevated cytokines, some of which may contribute to the growth and spread of cancer. It is not completely clear whether inflammation induced due to COVID-19 is chronic. In a case study of a male patient aged 72 years old who died from complications of COVID-19, a post-mortem biopsy showed diffuse alveolar damage with loose fibrous plugs [33]. Similarly, seven patients who died from SARS-CoV-2 infection in the same study showed diffuse alveolar damage with mild to moderate fibrosis present in the lung [33]. These findings suggest that excessive and persistent inflammation from viral infections could favor fibrotic lesions, increasing the risk of developing tumors with time. Another research based on the medical data of 51 admitted patients with different types of cancers who were positive for SARS-CoV-2 infection from April to May 2020 reported 11 patients with mild/moderate types of COVID-19 and 40 with severe COVID-19 infection [7]. Among 40 patients, 31 had comorbidities. Hypertension was the most common comorbidity followed by diabetes and chronic obstructive pulmonary disease.

Later cohort studies validated the earlier reports. In a large multicenter study from the USA, Canada and Spain, an association of increased mortality from COVID-19-infected patients was emphasized for those with a history of hypertension and cardiovascular disease. The study included 928 patients with cancer and COVID-19, and independent factors linked with death from coronavirus included age, male gender, smoking status and several comorbidities [34]. In addition, patients more susceptible to COVID-19 also had co-morbid cardiovascular conditions. Cardiovascular complications of COVID-19 overlap with those encountered during cancer treatment. The study noted that cardiovascular complications among patients with COVID-19 increased. This observation has implications for monitoring, the administration of chemotherapy, diagnosis and treatment of the disease during COVID-19 [34].

## 7. COVID-19 Infection, Cancer, and Healthcare System

The COVID-19 pandemic has affected cancer care, delivery of care and management of cancer patients, including various aspects such as patient assessment, diagnosis, therapies, treatments and surgeries. As the healthcare workforce was deployed in COVID-19 support, it has affected the delivery of care for other diseases in every aspect; for example, a study from Germany revealed that COVID-19 was the priority of the healthcare system, and the workforce was aligned to identify undiagnosed cases; thus, a significant drop in cancer diagnosis was observed in 2020 compared to 2019 [35]. An early cancer diagnosis is necessary to prevent the risk of development as it may react to end-stage if it remains untreated. Cancer develops in four stages, and 90% of patients with cancer who do not receive appropriate care and treatment rarely make it to five years. Therefore, diagnosis is necessary to prevent the development [17]. Another study described that COVID-19 had disrupted the healthcare system of both developed and developing countries, which led to a substantial reduction in cancer diagnosis and have increased backlog of cases that further increased the severity of illness and risk of mortality among those living with undiagnosed cases [36].

### 7.1. Challenges That Cancer Patients with COVID-19 Infection Face

The major challenge in managing cancer patients was the unavailability of hospital beds due to the changes in resource allocation and lack of cancer care support. Therefore, the risk remains high even if the treatment continues. In addition, healthcare workers responsible for the care of cancer patients must focus on COVID-19 patients because of the drastic increase of COVID-19 patients in hospitals [37]. Moreover, hospitals faced a severe shortage of medical resources during the COVID-19 pandemic, and many hospitals even decreased non-emergency admissions and services. Therefore, treating cancer patients on time was challenging, adversely affecting their prognosis [38]. Moreover, during the COVID-19 pandemic, screening for cancer decreased significantly; for instance, breast cancer screening dropped by 94% in 2020 [39]. Delays in cancer screening during the pandemic resulted in delayed diagnoses, leading to an increased number of patients diagnosed in an emergency setting and with late-stage cancers. Consequently, a delay in the effective treatment of cancer patients was observed [40].

The Patient-Reported Information Multidimensional Exploration (PRIME) framework is a validated tool that is used for the extraction, analysis and synthesis of patient-reported information on physical and mental well-being, such as symptoms, side effects, treatment decisions, real-life experiences, mental health issues, and the emotional burden of patients with cancer, cancer survivors and caregivers. PRIME was applied to detect the emotions and impact of media announcements of COVID-19 on the health and well-being of patients [41]. A report by Moraliyage (2021) presented the impact of COVID-19 on cancer care following the application of the PRIME framework that helped to understand the pulse of patients with cancer in terms of their healthcare expectations, information needs, mental needs and emotional well-being. These findings suggested that due to the exhausted healthcare resources, proper care was compromised, affecting cancer patients’ physical and mental well-being. The COVID-19 pandemic has also affected the quality of life (QoL) in patients with cancer. Patients during COVID-19 had lower scores of QoL. A cross-sectional study showed that 9% of participants with cancer had refrained from consulting the doctor due to the fear of COVID-19 infection, whilst 80% of patients reported their concern regarding the pandemic. Some patients tested positive, which further increased their fear.

### 7.2. Management of COVID-19 Infection in Cancer Patients

Individuals with a history of cancer are always at high risk of recurrence. Recurrence and increased severity are the most distressing for patients as they deal with uncertainty and require hospital visits for treatment. During the COVID-19 pandemic, patients have been asked to follow self-management and use telehealth services for all non-emergency cases. Telehealth or telemedicine enables patient and physician interaction using video conferencing platforms and secure messaging systems. Healthcare providers send a web link to patients to access their appointment. The telehealth meeting starts when the patient clicks on the provided link that guides the patient to reach his physician. During COVID-19, telemedicine was considered a convenient framework for cancer patient care. Table 1 summarizes the various studies on the effectiveness of telehealth in delivering patient care during the COVID-19 pandemic.

However, cancer patients still have to visit hospitals for regular check-ups, which is risky because of the increased risk of getting infected by COVID-19 [51]. The risk of COVID-19 for cancer patients affects the health professional and cancer care delivery. For example, during the COVID-19 pandemic, healthcare facilities prioritized infection and deployed their resources to combat it, consequently affecting care delivery for cancer and other chronic diseases. Therefore, health professionals, oncologists and nurses have faced the dilemma regarding chemotherapy, whether treatment should be continued or delayed and, as such, would affect the patient’s health. Many cancer patients have been infected with COVID-19, and it is recommended that chemotherapy should not be delayed; based on the risk vs. benefit analysis, the patient needs to be treated as a priority. As such, healthcare facilities continued to provide cancer treatment during the pandemic [52].

Apart from the therapies, the pandemic also impacted cancer-related surgeries due to the unavailability or shortage of personal protective equipment (PPE) in hospitals, lack of support for other diseases as a significant healthcare workforce was deployed for COVID-19 support, and risk due to shortage of beds and ventilators. To understand how the COVID-19 pandemic has affected cancer management, it is also essential to understand patients’ perspectives regarding their health during the pandemic. A cross-sectional revealed that cancer patients were more concerned about the short-term impact of cancer rather than knowing the impact of treatment [30]. Patient demographics and history of diseases are significant factors affecting cancer management. The study revealed that the risk of COVID-19 for cancer patients is considered moderate. However, individuals with a history of previously treated cancer or those undergoing cancer treatment reported a great fear of the virus [53]. Similarly, patients undergoing active treatment were concerned about their families as they were at risk of COVID-19. As discussed, healthcare facilities continued to provide care for cancer during the pandemic; however, some facilities have eased care delivery during a pandemic [54]. Almost 20% of individuals reported not having contact with their oncologists during the pandemic. Therefore, most studies support the hypothesis that COVID-19 has affected cancer care management [55].

## 8. Is the COVID-19 Vaccine Safe for Cancer Patients?

A single dose of the COVID-19 vaccine had shown weak and heterogeneous serological responses in haematological and solid malignancies. After receiving the second dose of the COVID-19 vaccine, seropositivity to the SARS-CoV-2 spike protein increased, but antibody titers remained low compared to healthy controls. It was also noted that seroconversion in haematological malignancies was significantly lower than in patients with solid tumors [56]. Similarly, in a study of 200 cancer patients, high seroconversion rates (94%) were noted in those who received both vaccine doses. In addition, patients with solid tumors showed seroconversion by 98% compared to hematologic malignancies (85%) [57].

A retrospective cohort study recruited 373 patients who received their first vaccine dose (Pfizer/BioNTech, Oxford/AstraZeneca, and Moderna). Among these patients, about 88.5% received anticancer treatment, and 76.1% developed vaccine-related adverse events such as sore arms, fatigue, and headaches [58]. A randomized, placebo-controlled, observer-blinded global phase 3 clinical trial included 3813 cancer patients. Four participants that received BNT162b2 and 71 placebos were infected with SARS-CoV-2 seven days after the second dose of the COVID-19 vaccine. The vaccine efficacy was 94.4% up to 6 months after the second dose follow-up, and vaccine efficacy was 91.1% in the overall trial population after the same follow-up. Therefore, the BNT162b2 vaccine has a similar efficacy and safety profile as the overall trial population [46,59]. COVID-19 vaccines are considered safe and well-tolerated in cancer patients. However, the seroconversion rate remains lower than the general population. Table 2 lists out various studies that were conducted on the effectiveness of COVID-19 vaccine among cancer pateints.

## 9. Conclusions

COVID-19 has been challenging because it affected overall health and also burdened healthcare systems across the globe. The significant challenge in managing cancer was the delays patients experienced in accessing healthcare or the lack of appropriate care, which directly affected their physical and mental well-being. During the COVID-19 pandemic, cancer patients have not received adequate treatment on time due to the unavailability of beds and healthcare workers. Consequently, the pandemic has affected the delivery of cancer care and increased the risk of mortality for patients. In addition, cancer patients are a high-risk population to be infected with COVID-19 because they are immunocompromised. Therefore, COVID-19 vaccines have been suggested and are considered safe and well-tolerated for cancer patients.

## Figures and Tables

**Table 1:** Effectiveness of different patient care frameworks among cancer patients during COVID-19.

Number of Patients	Framework	Study Type	Outcomes
145 cancer patients [42]	Telehealth: video conferencing and secure messaging system	Cross-sectional, web-based survey	Participants generally reported positive satisfaction with each platform. Patients were more satisfied with secure messaging because they could ask questions without scheduling an appointment.
240 cancer patients [43]	Telemedicine: a phone or video-conferencing service to provide medical care remotely	A retrospective cohort study	Telemedicine provided a timely delivery of oncologic care during the initial wave of the COVID-19 pandemic.
379 cancer patients [44]	Telemedicine: delivery of health services using communication technology by defeating boundaries of geographic and socioeconomic distances to improve access to care.	Questionnaires	Telemedicine can improve cancer care delivery, especially for those who live far away from health centers.
172 cancer patients [45]	Telemedicine: a platform for bridging health services and medical care between physicians and patients	Interview-based questionnaires	Telemedicine is identified as safe and effective, and patients did not feel that it affected medical care or the patient–physician relationship.
77 cancer patients [46]	Telemedicine is operated through security-certified digital platforms.	A retrospective cohort study	Telemedicine appointment is an effective tool to minimize patient exposure to clinics or hospital environment infected with COVID-19.
421 cancer patients. The majority of patients had breast cancer [47]	Telemedicine: communication methods were video calling, WhatsApp or short messages and voice call.	Cross-sectional study	The demands of 92.8% of patients using Telemedicine were fulfilled, and about 93.0% of patients living in different states were satisfied as they didn’t have to travel to the hospital.
1077 cancer patients [48]	Telemedicine: telephone only or interactive audio-visual capabilities using (smartphones or tablets/laptops/desktop computers)	Survey	45% of patients preferred Telemedicine, and 34% preferred office visits. Among the survey, respondent patients were highly satisfied and preferred Telemedicine.
83 patients with lung cancer [49]	Telemedicine: patients were contacted via video calls, and patients sent documents to the physician via WeTransfer link or email	Retrospective study	76.5% of patients preferred video consultation services. It is considered better than or comparable to an in-person visit. This study suggests continuing Telemedicine even after the COVID-19 pandemic.
45 cancer patients [50]	Telemedicine: intervention was provided through video conference via Zoom, Google Hangouts video or WhatsApp video chat.	Intervention study	This study indicated that providing a supportive and palliative care telemedicine program to cancer patients during COVID-19 in resource-limited settings, including low-income and middle-income countries was the possible and effective way.

**Table 2:** Impact of COVID-19 vaccine on cancer patients.

Number of Patients	Vaccine	Study Type	Outcomes
A total of 184,485 Veteran’s Affairs patients with cancer patients were included; among them, 113,796 were vaccinated [60]	SARS-CoV-2 vaccine	A retrospective, multicentre, nationwide cohort study	SARS-CoV-2 infections were found in 436 patients, among which 161 were vaccinated, and 275 were unvaccinated. Seventeen deaths were in the vaccinated group, and twenty-seven were in the unvaccinated group due to COVID-19. Patients who received chemotherapy endocrine therapy within 3 months before the first vaccine dose were shown to have vaccine effectiveness by 57% and 76%, respectively, and 85% for those who had not received systemic therapy for at least 6 months before taking the vaccine.
326 patients with solid tumours treated with anticancer medications [61]	2 doses of the BNT162b2 vaccine	Retrospective study	Immunoglobulin G titers were significantly lower among cancer patients compared to control. Thirty-nine cancer patients were seronegative compared to the control group. No side effect of the vaccine was observed.
621 cancer patients and 256 controls [62]	Two doses of BNT162b2, two doses of mRNA-1273, or one dose of Ad26.COV2.S	Meta-analysis	Cancer patients with solid tumours reported having >90% antibody responses, while antibody titers were significantly lower than healthy participants. Seroconversion rate was lower in patients with hematologic malignancies. COVID-19 vaccines were shown to have a good safety profile with no grade 3–4 adverse events.
140 patients with solid malignancies and 215 without cancer [63]	Two doses of BNT162b2	Cross-sectional study	The humoral response in the cancer patient group was significantly (*p* < 0.001) lower than in the non-cancer group, including seronegative, by 14.3% and 1.4%, respectively. Median IgG levels by 2231 AU/mL and 4100 AU/mL, respectively.
151 cancer patients and 54 healthy controls participants [64]	BNT162b2 vaccine	Prospective observational study	18 of 19 patients with solid cancer, all 12 healthy controls, and 3 of 5 patients with haematological cancers were seropositive compared with 10 of 33, 18 of 21, and 4 of 36, respectively, who did not receive a booster. The vaccine has shown to be well tolerated, and no vaccine-related deaths were observed.

## Data Availability

No new data were created or analyzed in this study. Data sharing does not apply to this article.
